# Genome Sequences of West Nile Virus Reference Materials

**DOI:** 10.1128/MRA.00740-21

**Published:** 2021-10-28

**Authors:** Hanna Roth, Julia Kreß, Michael Chudy, Johannes Blümel, Jonas Schmidt-Chanasit, Dániel Cadar, Matthias Niedrig, Emőke Ferenczi, Maike Herrmann, Sally A. Baylis, Csaba Miskey

**Affiliations:** a Paul-Ehrlich-Institut, Langen, Germany; b Bernhard Nocht Institute for Tropical Medicine, Hamburg, Germany; c Robert Koch Institute, Berlin, Germany; d National Center for Epidemiology, Budapest, Hungary; KU Leuven

## Abstract

We report the sequences of two West Nile virus (WNV) strains (lineages 1 and 2) developed by the Paul-Ehrlich-Institut as reference materials. The materials are calibrated against the 1^st^ World Health Organization WNV RNA International Standard and are intended for use in nucleic acid technology assays supporting transfusion safety.

## ANNOUNCEMENT

West Nile virus (WNV) is a *Flavivirus* (family *Flaviviridae*) transmitted by *Culex* mosquitoes and causing infections in birds, horses, and humans (1). First isolated from a Ugandan patient in 1937 (2), WNV subsequently spread within Africa, Asia, the Middle East, North America, and Europe (3). Typically, WNV infections are asymptomatic; however, some individuals develop West Nile fever and occasionally neuroinvasive disease (4). With the expansion of WNV in Europe, including Germany (5), transmission by blood transfusion is a concern, and implementation of nucleic acid amplification technique (NAT)-based donor screening is necessary once human cases become endemic and for travelers returning from affected areas (6). To support testing by transfusion services and NAT assay developers, reference materials were prepared by the Paul-Ehrlich-Institut for WNV lineages 1 (NY99; flamingo) and 2 (Héja; goshawk), reflecting circulating European clades (7, 8). The isolates were passaged once in Vero E6 cells and heat-inactivated as previously described (9); no infectivity was detected following heat inactivation. Heat-inactivated stocks were diluted in human plasma, dispensed into vials, and lyophilized; batches of reference material prepared from NY99 and Héja were designated 13299/19 and 13300/19, respectively. RNA was extracted using the ExiPrep Dx viral RNA kit (Bioneer Corp., Daejeon, Republic of Korea) (10). Libraries were prepared using a modified version of the “not not so random priming” method (11). Following cDNA synthesis, barcoded Illumina libraries were prepared by PCR amplification using NEBNext Ultra II master mix (New England Biolabs, Frankfurt, Germany); amplicons were recovered and sequenced using a MiSeq instrument with the paired-end (2 × 300-bp) setting as previously described (12).

Majority consensus sequences were generated from the processed and mapped reads based on the reference sequences (13); default parameters were applied unless otherwise stated. The sequencing statistics are shown in Table 1. Fastp v0.20.0 (14) was used for quality trimming and adapter removal. After quality control, the reads were mapped using BWA-MEM v0.7.12-r1039 (15). Host-derived sequences (Chlorocebus sabeus; GenBank accession number GCA_000409795.2) were removed by specifying the minimum seed length (-k 31). Unmapped reads were extracted using SAMtools v1.7 (16) and bamtofastq v2.17.0 (17) and subsequently mapped to the WNV reference genomes submitted under GenBank accession numbers AF196835.2 (lineage 1) or DQ116961.1 (lineage 2). Host-free alignments were deduplicated using MarkDuplicates in the Picard toolkit (http://broadinstitute.github.io/picard) and left-aligned using LeftAlignIndels in GATK v4.0 (18) Variant calling was performed using LoFreq v2.1.3 (19). The sequence determined for isolate NY99 was 11,025 bp long, with seven nucleotide changes (all synonymous) compared to the prototype (AF196835.2). The Héja isolate, 11,028 bp long, is closely related to viruses isolated from goshawks in Central Europe, confirming its position within the Central European lineage 2 clade. Héja showed 27 nucleotide differences to DQ116961.1 (>99% identity), resulting in 6 amino acid changes (3 nonsynonymous). The Héja virus has not always been adequately detected in external quality assessment programs (8); therefore, knowledge of the sequence is important for improving assays to ensure detection of similar viruses going forward.

**TABLE 1:** West Nile Virus NY99 and Héja sequencing statistics

Parameter^*a*^	Data for isolate:	
	NY99	Héja
GenBank accession no.	MZ605381	MZ605382
BioProject accession no.	PRJNA759393	PRJNA759393
WNV lineage	1	2
PEI reference material code no.^*b*^	13299/19	13300/19
Length (bp)	11,025	11,028
Potency	6.25 log_10_ IU/ml	5.88 log_10_ IU/ml
No. of reads		
Raw reads	2,624,302	3,084,270
After QC	2,530,266	2,979,944
After removal of host sequences	461,663	447,665
Total length of reads (bp)	94,234,259	89,678,647
Avg read length (bp)	204	200
No. of mapped reads	350,568	351,140
Proportion mapped		
% of raw reads	13.4	11.4
% of QC reads	13.9	11.8
% of reads after removal of host sequences	75.9	78.4
Mean depth of coverage (×)	6,522	6,497

a QC, quality control.

b PEI, Paul-Ehrlich-Institut.

Both reference materials are calibrated against the World Health Organization International Standard for WNV for NAT-based assays (20) and are considered “secondary standards” (21).

### Data availability.

The sequences of strains NY99 and Héja reported here have been deposited in GenBank under the accession numbers MZ605381 and MZ605382, respectively. The sequencing read data have been deposited in the NCBI SRA under accession number PRJNA759393. The reference materials are available from the Paul-Ehrlich-Institut (https://www.pei.de).
